# The aminoglycoside G418 hinders *de novo* prion infection in cultured cells

**DOI:** 10.1016/j.jbc.2021.101073

**Published:** 2021-08-12

**Authors:** Hamza Arshad, Zeel Patel, Mohadeseh Mehrabian, Matthew E.C. Bourkas, Zaid A.M. Al-Azzawi, Gerold Schmitt-Ulms, Joel C. Watts

**Affiliations:** 1 Tanz Centre for Research in Neurodegenerative Diseases, University of Toronto, Toronto, Ontario, Canada; 2 Department of Biochemistry, University of Toronto, Toronto, Ontario, Canada; 3 Department of Laboratory Medicine and Pathobiology, University of Toronto, Toronto, Ontario, Canada

**Keywords:** prion, cell culture, protein misfolding, protein aggregation, neurodegeneration, antibiotics, DAPI, diamidino-2-phenylindole, GPI, glycosylphosphatidylinositol, HaPrP, hamster PrP, IRES, internal ribosome entry site, MoPrP, mouse PrP, NCAM, neural cell adhesion molecule, PBS, phosphate-buffered saline, PK, proteinase K, PrP, prion protein, RT-QuIC, real-time quaking-induced conversion, TBS, Tris buffered saline, ThT, Thioflavin T

## Abstract

The study of prions and the discovery of candidate therapeutics for prion disease have been facilitated by the ability of prions to replicate in cultured cells. Paradigms in which prion proteins from different species are expressed in cells with low or no expression of endogenous prion protein (PrP) have expanded the range of prion strains that can be propagated. In these systems, cells stably expressing a PrP of interest are typically generated *via* coexpression of a selectable marker and treatment with an antibiotic. Here, we report the unexpected discovery that the aminoglycoside G418 (Geneticin) interferes with the ability of stably transfected cultured cells to become infected with prions. In G418-resistant lines of N2a or CAD5 cells, the presence of G418 reduced levels of protease-resistant PrP following challenge with the RML or 22L strains of mouse prions. G418 also interfered with the infection of cells expressing hamster PrP with the 263K strain of hamster prions. Interestingly, G418 had minimal to no effect on protease-resistant PrP levels in cells with established prion infection, arguing that G418 selectively interferes with *de novo* prion infection. As G418 treatment had no discernible effect on cellular PrP levels or its localization, this suggests that G418 may specifically target prion assemblies or processes involved in the earliest stages of prion infection.

Prion diseases are a group of fatal neurodegenerative disorders caused by the accumulation of toxic protein aggregates in the brain (1, 2). Examples of prion diseases include chronic wasting disease in cervids, scrapie in sheep, “mad cow” disease, and Creutzfeldt–Jakob disease in humans. Prions, which were originally defined as proteinaceous infectious particles (3), arise from the structural rearrangement of the host-encoded prion protein (PrP) (4). In its native state, the cellular prion protein (PrP^C^) has a predominantly α-helical structure and is attached to the outer leaflet of the cell membrane of neurons and other central nervous system cells *via* a glycosylphosphatidylinositol (GPI) anchor (5, 6). PrP^C^ has been implicated in a wide range of functions, specifically in regard to myelin maintenance in the peripheral nervous system and regulation of neural cell adhesion molecule (NCAM) polysialylation (7, 8). During disease, PrP^C^ undergoes a profound conformational rearrangement to form β-sheet-rich aggregates that are protease-resistant, neurotoxic, and infectious (9). This conformer is termed PrP^Sc^, and its infectious nature allows it to template the conversion of PrP^C^ into additional copies of PrP^Sc^. Therefore, PrP^Sc^ functions as a self-propagating protein assembly, allowing for the accumulation and spread of PrP^Sc^ within the brain. This templated conversion process is believed to be the central event in the pathogenesis of prion disease.

Animal bioassays are currently the gold standard for assessing prion transmissibility and studying the pathogenesis of prion disease (10, 11). However, animal experiments are often long and costly, and therefore cellular models that can recapitulate the templated conversion of PrP^C^ to PrP^Sc^ have provided a cost-efficient paradigm for investigating the biology associated with prion disease (12). Several different lines of immortalized cells expressing murine PrP^C^ have been identified that can become infected with mouse prions (13, 14, 15, 16, 17, 18, 19). Perhaps the most important use for these cellular models is the platform they provide for identifying small molecule inhibitors of prion replication (20). A high-throughput screen conducted in prion-infected N2a neuroblastoma cells yielded several 2-aminothiazole compounds, including IND24, which could reduce levels of proteinase K (PK)-resistant PrP (PrP^res^) in cells and significantly increase the survival of prion-infected mice (21, 22). However, while these compounds displayed activity against mouse prions in cultured cells and mice, they were ineffective at inhibiting the replication of human prions in transgenic mice expressing human PrP (22, 23, 24, 25). Thus, antiprion compounds need to be identified and validated using a paradigm capable of replicating the exact type of prions that the drug is intended to treat.

The availability of cellular paradigms for replicating nonmouse prions is comparatively limited. Very few PrP^C^-expressing cell lines from nonmouse species that can become infected with species-matched prions have been identified (20, 26, 27, 28). A more fruitful approach has been to take a cell line that does not express detectable levels of PrP^C^ and then engineer it to express PrP^C^ from the desired species. For instance, rabbit RK13 cells become susceptible to chronic wasting disease and scrapie prions upon expression of elk or sheep PrP^C^, respectively (29, 30, 31). Similar paradigms have been developed using cells derived from PrP knockout mice or gene-edited PrP^C^-null (PrP^−/−^) cell lines as a starting point (32, 33, 34, 35, 36). In each of these cases, selection of stably transfected cells expressing the desired PrP^C^ is typically accomplished by coexpression of an antibiotic resistance gene and subsequent antibiotic treatment. A commonly used antibiotic for selection of stably transfected cells is G418 (37), also known as Geneticin, which is an aminoglycoside originally identified in the bacterium *Micromonospora rhodorangea*. G418 binds to the ribosome and inhibits translational elongation in both prokaryotic and eukaryotic cells, thus interfering with protein synthesis and cell growth (38). Resistance to G418 is conferred *via* expression of phosphotransferase enzymes such as neomycin phosphotransferase II (39).

During our attempts to study prion propagation using gene-edited PrP^−/−^ cells that had been stably transfected with vectors encoding PrP^C^, we made the surprising discovery that G418 interferes with *de novo* prion infection in cultured cells. In this study, we investigate the inhibitory role of G418 on prion infection using different cell lines and prion strains. Our findings suggest that to ensure optimal results, strategies to avoid or limit the use of G418 during prion infection experiments should be implemented.

## Results

### G418 inhibits *de novo* prion infection in cultured cells

**Figure 1:**
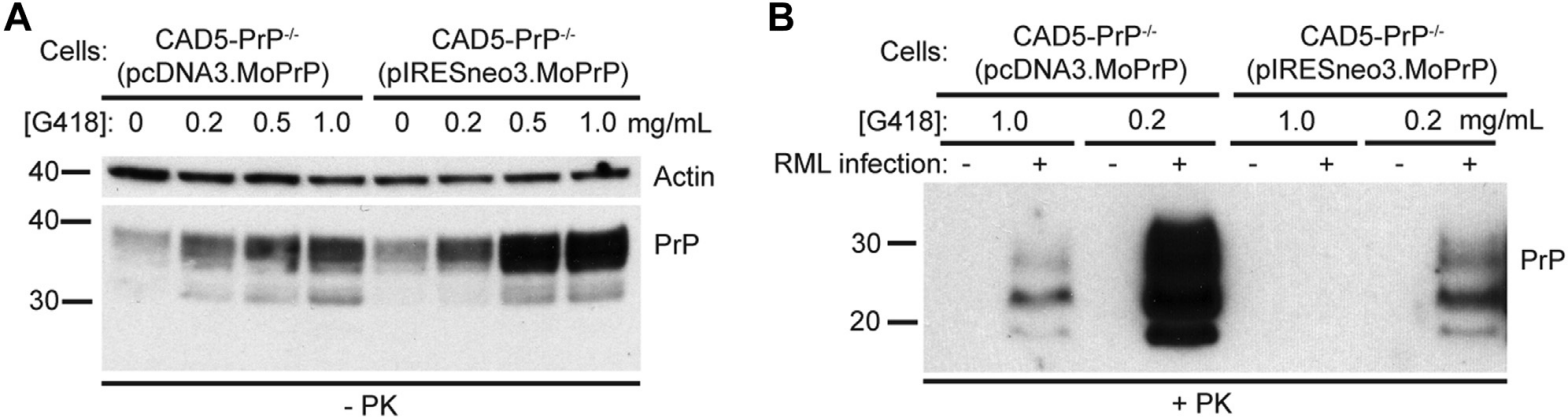
**Increasing levels of G418 promote expression of plasmid-encoded mouse PrP in PrP**^**−/−**^**cells yet lead to lower PrP**^**res**^**levels following RML infection.***A*, immunoblot of PrP^C^ levels in lysates from uninfected CAD5-PrP^−/−^(pcDNA3.MoPrP) and CAD5-PrP^−/−^(pIRESneo3.MoPrP) cells cultured in the presence of the indicated concentrations of G418 for three passages. *B*, immunoblot of proteinase K (PK)-resistant PrP (PrP^res^) levels in CAD5-PrP^−/−^(pcDNA3.MoPrP) and CAD5-PrP^−/−^(pIRESneo3.MoPrP) cells after three passages following infection with mouse RML prions in the presence of the indicated concentrations of G418. PrP^res^ was detected using HuM-P, whereas PrP^C^ was detected with the antibody HuM-D13. In panel *A*, the blot was reprobed with an actin antibody to assess equal protein loading. Molecular weight markers indicate kDa.

Murine CAD5 cells, which are derived from the catecholaminergic line Cath.a (40, 41) and express mouse PrP (MoPrP), are capable of replicating many different mouse prion strains, including RML, Me7, and 22L (16, 22, 42). We recently generated CAD5-PrP^−/−^ cells lacking endogenous MoPrP and found that, following stable expression of hamster PrP (HaPrP), they can become infected with several strains of hamster prions (34). In parallel, we generated polyclonal pools of CAD5-PrP^−/−^ cells stably expressing MoPrP [CAD5-PrP^−/−^(MoPrP) cells] using two different vectors (pcDNA3 and pIRESneo3), both of which confer resistance to the selectable agent G418. G418 is typically used at concentrations of 1.0 and 0.2 mg/ml for selection and maintenance of stably transfected cell lines, respectively. For both lines of CAD5-PrP^−/−^(MoPrP) cells, maintenance of the cells in higher concentrations of G418 resulted in higher levels of PrP^C^ expression suggesting that, in the absence of selective agent, the composition of these polyclonal pools of cells drifts toward cells with lower levels of PrP^C^ expression (Fig. 1*A*). When CAD5-PrP^−/−^(MoPrP) cells were challenged with RML prions in the presence of 0.2 mg/ml G418, successful prion infection was observed, as indicated by the presence of PrP^res^ in cellular lysates (Fig. 1*B*). Paradoxically, when 1.0 mg/ml G418 was used in an attempt to increase PrP^C^ expression levels and thus maximize PrP^res^ production (43), the efficiency of RML prion infection in CAD5-PrP^−/−^(MoPrP) cells was substantially reduced (Fig. 1*B*). This led us to hypothesize that G418 may interfere with *de novo* prion infection in cultured cells.

**Figure 2:**
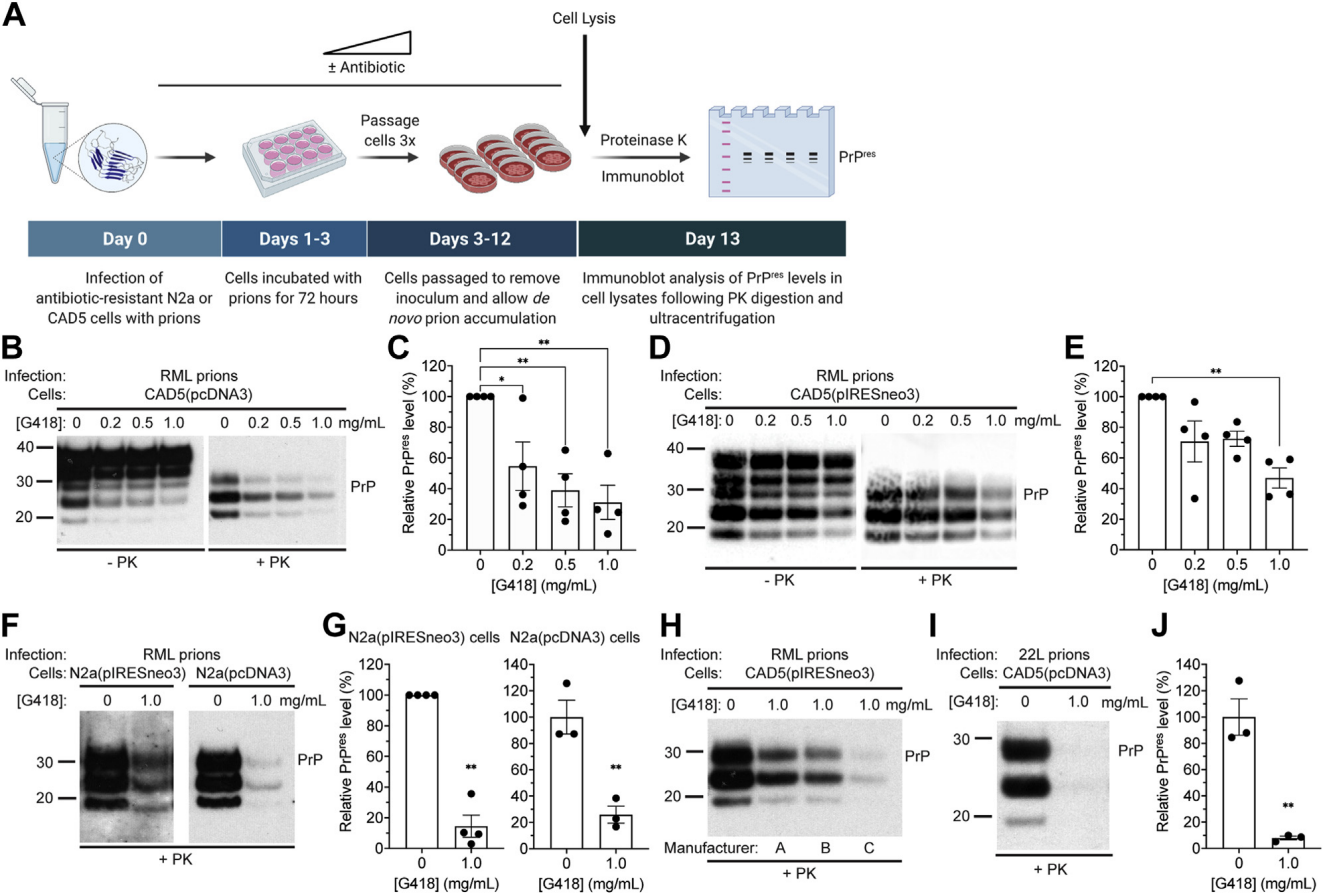
**G418 inhibits *de novo* prion infection in cultured cells expressing endogenous mouse PrP.***A*, schematic of the experimental workflow for infection of antibiotic-resistant cell lines with prions. All infection experiments were analyzed following three passages. *B*, immunoblots of total PrP (−PK) and PrP^res^ (+PK) levels in CAD5(pcDNA3) cells following infection with RML prions in the presence of the indicated concentrations of G418. *C*, quantification of PrP^res^ levels in CAD5(pcDNA3) cells infected with RML prions in the presence of increasing concentrations of G418. n = 4 independent biological replicates (data is mean ± SEM). ∗*p* = 0.034 for 0.2 mg/ml, ∗∗*p* = 0.0055 for 0.5 mg/ml, and ∗∗*p* = 0.0023 for 1.0 mg/ml G418 by one-way ANOVA followed by Dunnett’s multiple comparisons test. *D*, immunoblots of total PrP and PrP^res^ levels in CAD5(pIRESneo3) cells following infection with RML prions in the presence of the indicated concentrations of G418. *E*, quantification of PrP^res^ levels in CAD5(pIRESneo3) cells infected with RML prions in the presence of G418. n = 4 independent biological replicates (data is mean ± SEM). ∗∗*p* = 0.0039 compared with infections performed in the absence of G418 by one-way ANOVA followed by Dunnett’s multiple comparisons test. *F*, immunoblots of PrP^res^ levels in N2a(pIRESneo3) and N2a(pcDNA3) cells following infection with RML prions in the absence or presence of 1 mg/ml G418. *G*, quantification of PrP^res^ levels (data is mean ± SEM) in N2a(pIRESneo3) and N2a(pcDNA3) cells infected with RML prions in the presence or absence of G418 (n = 4 or 3 independent biological replicates, respectively). For N2a(pIRESneo3) cells, ∗∗*p* = 0.0013 by a one sample *t* test. For N2a(pcDNA3) cells, ∗∗*p* = 0.0067 by a two-tailed unpaired *t* test. *H*, immunoblot of PrP^res^ levels in CAD5(pIRESneo3) cells following infection with RML prions in the absence or presence of 1 mg/ml G418 sourced from three different manufacturers. *I*, immunoblot of PrP^res^ levels in CAD5(pcDNA3) cells following infection with 22L prions in the absence or presence of 1 mg/ml G418. *J*, quantification of PrP^res^ levels (data is mean ± SEM) in CAD5(pcDNA3) cells infected with 22L prions in the presence or absence of G418 (n = 3 independent biological replicates). ∗∗*p* = 0.0027 by a two-tailed unpaired *t* test. In *B*, *D*, *F*, *H*, and *I*, undigested PrP blots were probed with the antibody HuM-D13, whereas blots of PrP^res^ were probed with HuM-P. Molecular weight markers indicate kDa.

To test the effect of G418 on RML prion infection, we stably transfected wild-type CAD5 cells with two different empty vectors that confer G418 resistance to generate CAD5(pcDNA3) and CAD5(pIRESneo3) cells. These G418-resistant cells, which express endogenous levels of MoPrP, were then treated with RML prions in the presence of increasing concentrations of G418 (Fig. 2*A*). Following prion exposure, cells were passaged three times to allow for complete removal of the inoculum by dilution and the emergence of *de novo* RML prion-infected cells. In CAD5(pcDNA3) cells, increasing concentrations of G418 resulted in a dose-dependent reduction in PrP^res^ levels obtained following challenge with RML prions (Fig. 2, *B* and *C*). Similar results were obtained when CAD5(pIRESneo3) cells were used (Fig. 2, *D* and *E*). To analyze whether this effect was specific to CAD5 cells, we generated G418-resistant murine N2a neuroblastoma cells by stably transfecting them with either pcDNA3 or pIRESneo3 and then challenged them with RML prions in the presence or absence of G418. N2a cells are widely used for studying prion propagation but are susceptible to a more limited range of mouse prion strains than CAD5 cells (14, 16). As with CAD5 cells, the presence of G418 significantly decreased PrP^res^ levels in N2a(pcDNA3) and N2a(pIRESneo3) cells following RML prion infection (Fig. 2, *F* and *G*). Preparations of G418 obtained from three different manufacturers interfered with RML prion infection in CAD5(pIRESneo3) cells (Fig. 2*H*). Moreover, this effect was not specific to the RML strain since G418 also hindered infection of CAD5(pcDNA3) cells with the 22L strain of mouse prions (Fig. 2, *I* and *J*). Thus, G418 obstructs *de novo* prion infection in cells challenged with mouse prion strains.

**Figure 3:**
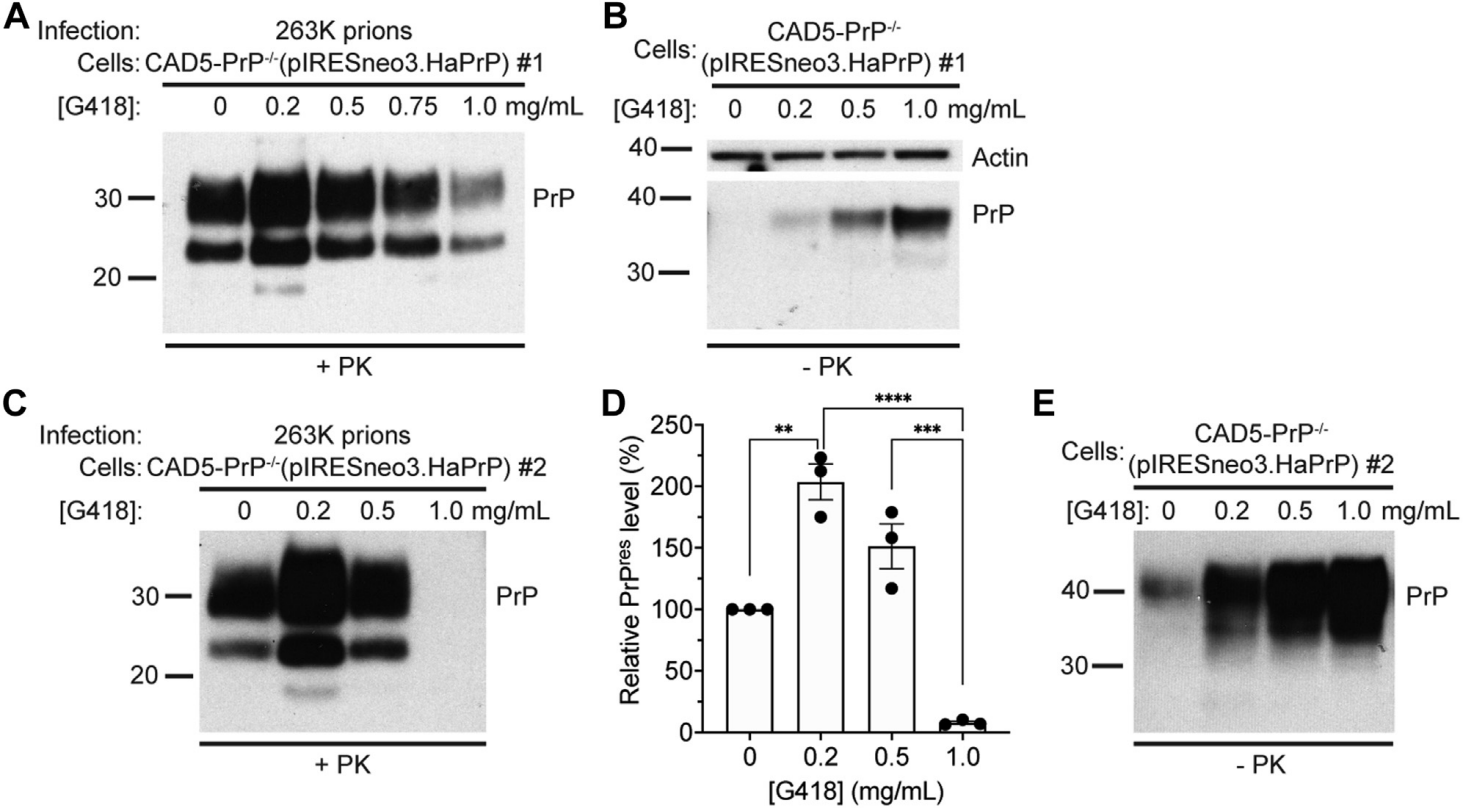
**G418 inhibits infection of cultured PrP**^**−/−**^**cells expressing hamster PrP with hamster prions.***A*, immunoblot of PrP^res^ levels in CAD5-PrP^−/−^(pIRESneo3.HaPrP) cells (line #1) after three passages following infection with hamster 263K prions in the presence of the indicated concentrations of G418. *B*, immunoblot of PrP^C^ levels in uninfected CAD5-PrP^−/−^(pIRESneo3.HaPrP) cells (line #1) cultured in the presence of the indicated concentrations of G418 for three passages. *C*, immunoblot of PrP^res^ levels in an independent line of CAD5-PrP^−/−^(pIRESneo3.HaPrP) cells (line #2) after three passages following infection with hamster 263K prions in the presence of the indicated concentrations of G418. *D*, quantification of PrP^res^ levels in CAD5-PrP^−/−^(pIRESneo3.HaPrP) cells (line #2) infected with 263K prions in the presence of increasing concentrations of G418. n = 3 independent biological replicates (data is mean ± SEM). ∗∗*p* = 0.0011, ∗∗∗*p* = 0.00011, and ∗∗∗∗*p* = 0.000011 by one-way ANOVA followed by Tukey’s multiple comparisons test. *E*, immunoblot of PrP^C^ levels in uninfected CAD5-PrP^−/−^(pIRESneo3.HaPrP) cells (line #2) cultured in the presence of the indicated concentrations of G418 for three passages. PrP^res^ was detected using HuM-P whereas PrP^C^ was detected with the antibody HuM-D13. In panel *B*, the blot was reprobed with an actin antibody to assess equal protein loading. Molecular weight markers indicate kDa.

We next asked whether G418 interferes with infection of cultured cells with a nonmouse prion strain, the 263K strain of hamster prions. For this purpose, we used CAD5-PrP^−/−^ cells that had been transfected with a vector that encodes HaPrP and confers resistance to G418 (pIRESneo3.HaPrP). We expanded these cells in the presence of G418 to generate a polyclonal pool of stably transfected cells [CAD5-PrP^−/−^(HaPrP) cells] (34). Exposure of these cells to 263K prions in the presence of a high concentration of G418 hindered prion infection compared with cells that were treated with a low concentration of G418 (Fig. 3*A*). Infection of cells in the presence of 0.2 mg/ml G418 resulted in higher levels of PrP^res^ than in cells that were infected in the absence of G418. We speculate that this is because, similar to what we observed in polyclonal CAD5-PrP^−/−^(MoPrP) cells (Fig. 1*A*), higher PrP^C^ levels were found in CAD5-PrP^−/−^(HaPrP) cells maintained in the presence of G418 (Fig. 3*B*). We repeated these experiments using an independent polyclonal line of CAD5-PrP^−/−^(HaPrP) cells and obtained similar results (Fig. 3, *C*–*E*). Thus, G418 interferes with the infection of cultured cells with mouse or hamster prion strains, regardless of whether PrP is expressed endogenously or *via* a vector-based heterologous promoter.

### G418 does not affect prion propagation in cells with established prion infection

**Figure 4:**
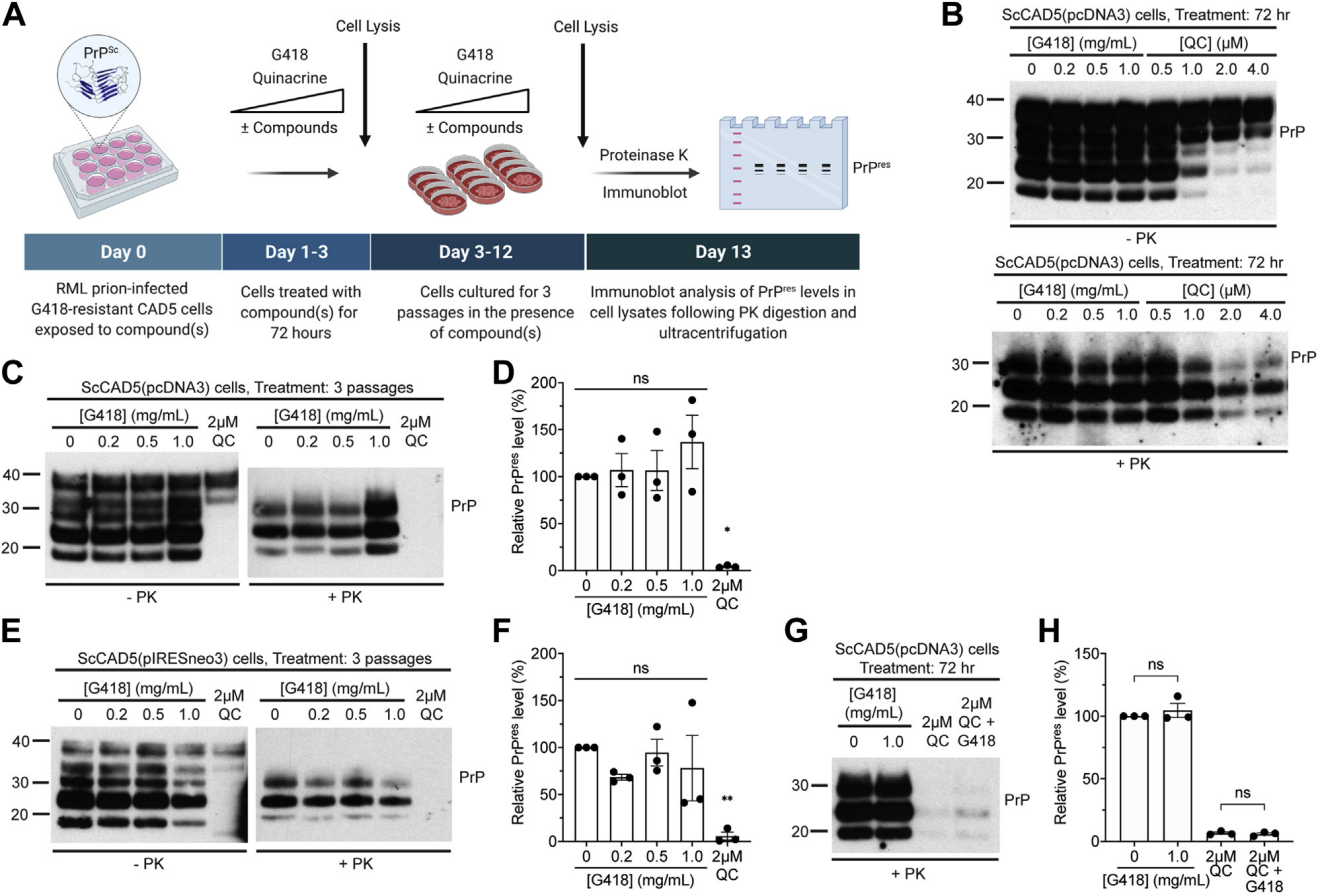
**Treatment of RML prion-infected cells with G418 has minimal effects on prion propagation.***A*, schematic of the experimental workflow for treatment of prion-infected G418-resistant cells with G418 or quinacrine (QC). PrP^res^ levels in cell lysates were analyzed following treatment for either 72 h or three passages. *B*, immunoblots of total PrP (−PK) and PrP^res^ (+PK) levels in RML prion-infected CAD5(pcDNA3) cells [ScCAD5(pcDNA3)] treated with the indicated concentrations of G418 or QC for 72 h. *C*, immunoblots of total PrP and PrP^res^ levels in ScCAD5(pcDNA3) cells following three passages in the presence of the indicated concentrations of G418 or 2 μM QC. *D*, quantification of PrP^res^ levels in ScCAD5(pcDNA3) cells following three passages in the presence of increasing concentrations of G418 or 2 μM QC. n = 3 independent biological replicates (data is mean ± SEM). ∗*p* = 0.011 compared with untreated cells by one-way ANOVA followed by Dunnett’s multiple comparisons test; ns, not significant. *E*, immunoblots of total PrP and PrP^res^ levels in ScCAD5(pIRESneo3) cells following three passages in the presence of the indicated concentrations of G418 or 2 μM QC. *F*, quantification of PrP^res^ levels in ScCAD5(pIRESneo3) cells following three passages in the presence of increasing concentrations of G418 or 2 μM QC. n = 3 independent biological replicates (data is mean ± SEM). ∗∗*p* = 0.0093 compared to untreated cells by one-way ANOVA followed by Dunnett’s multiple comparisons test; ns, not significant. *G*, immunoblot of PrP^res^ levels in ScCAD5(pcDNA3) cells treated with the indicated concentrations of G418, 2 μM QC, or 2 μM QC + 1.0 mg/ml G418 for 72 h. *H*, quantification of PrP^res^ levels in ScCAD5(pcDNA3) cells following treatment with G418, QC, or QC + G418 for 72 h. n = 3 independent biological replicates (data is mean ± SEM). PrP^res^ levels were not significantly different (*p* = 0.99) in cells treated with 2 μM QC or 2 μM QC + 1.0 mg/ml G418 as determined by one-way ANOVA followed by Tukey’s multiple comparisons test. In *B*, *C*, *E*, and *G* undigested PrP blots were probed with the antibody HuM-D13, whereas blots of PrP^res^ were probed with HuM-P. Molecular weight markers indicate kDa.

Having demonstrated that the presence of G418 at the time of prion infection reduces the accumulation of PrP^res^ in both CAD5 and N2a cells, we next asked whether G418 is able to modulate established prion infection in cultured cells. G418-resistant CAD5(pcDNA3) and CAD5(pIRESneo3) cells were infected with RML prions in the absence of G418 to generate ScCAD5(pcDNA3) and ScCAD5(pIRESneo3) cells and then treated with increasing concentrations of G418 (Fig. 4*A*). As a positive control, cells were treated with quinacrine, a small molecule known to reduce PrP^res^ levels in RML-infected N2a and CAD5 cells (22, 44). After a single 72-h treatment, G418 treatment did not reduce levels of PrP^res^ in ScCAD5(pcDNA3) cells, whereas quinacrine reduced PrP^res^ levels in a dose-dependent fashion (Fig. 4*B*). The treatment duration was then extended to encompass three passages (12–15 days of treatment) in the presence of varying doses of G418. After three passages, RML infection was cured in quinacrine-treated cells, whereas G418-treated ScCAD5(pcDNA3) and ScCAD5(pIRESneo3) cells showed no consistent reduction in PrP^res^ levels (Fig. 4, *C*–*F*). To check whether G418 may only be effective at reducing prion infection in cells with lower levels of PrP^res^, as may occur during the initial stages of *de novo* prion infection, we simultaneously treated ScCAD5(pcDNA3) cells for 72 h with both quinacrine (to lower PrP^res^ levels) and G418. We observed no additional reduction of PrP^res^ in cells treated with both quinacrine and G418 (Fig. 4, *G* and *H*), even when a lower concentration of quinacrine was used (Fig. S1). Collectively, these results suggest that G418 does not have a major effect on PrP^res^ levels in cells with established prion infection.

### Streptomycin, but not puromycin, inhibits *de novo* prion infection

**Figure 5:**
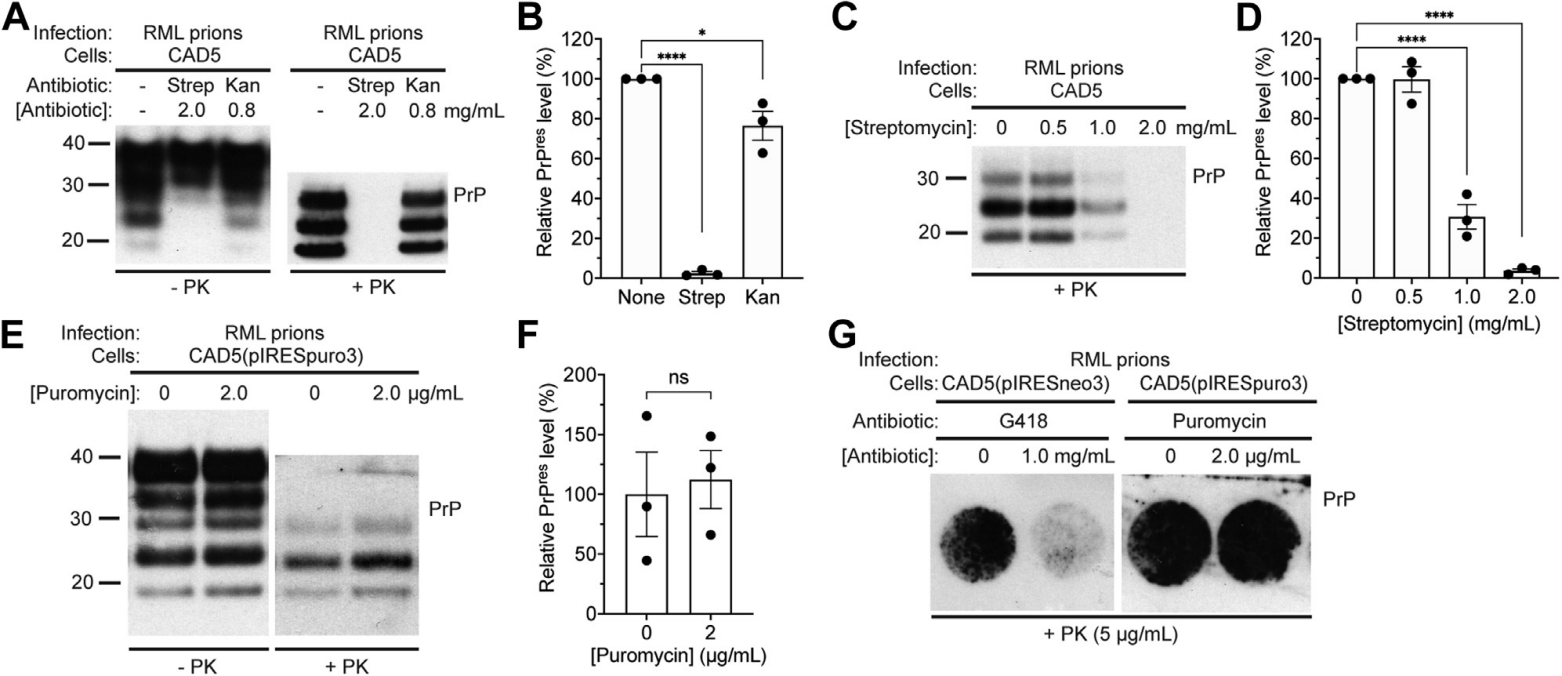
**The effects of aminoglycoside and aminonucleoside antibiotics on *de novo* infection of cultured cells with RML prions.***A*, immunoblots of total PrP (−PK) and PrP^res^ (+PK) levels in CAD5 cells after three passages following challenge with RML prions in the presence of either 2.0 mg/ml streptomycin (Strep) or 0.8 mg/ml kanamycin (Kan). *B*, quantification of PrP^res^ levels (data is mean ± SEM) in CAD5 cells infected with RML prions in the presence or absence of 2.0 mg/ml streptomycin or 0.8 mg/ml kanamycin (n = 3 independent biological replicates). ∗*p* = 0.014 and ∗∗∗∗*p* < 0.0001 compared with untreated cells by one-way ANOVA followed by Dunnett’s multiple comparisons test. *C*, immunoblot of PrP^res^ levels in CAD5 cells at passage three following challenge with RML prions in the presence of the indicated concentrations of streptomycin. Streptomycin is commonly added to cell media at a concentration of 0.1 mg/ml to discourage microbial growth. *D*, quantification of PrP^res^ levels (data is mean ± SEM) in CAD5 cells infected with RML prions in the presence of the indicated concentrations of streptomycin (n = 3 independent biological replicates). ∗∗∗∗*p* < 0.0001 compared with untreated cells by one-way ANOVA followed by Dunnett’s multiple comparisons test. *E*, immunoblots of total PrP and PrP^res^ levels in CAD5(pIRESpuro3) cells at passage three following challenge with RML prions in the absence or presence of 2 μg/ml puromycin. *F*, quantification of PrP^res^ levels in CAD5(pIRESpuro3) cells infected with RML prions in the presence or absence of puromycin (n = 3 independent biological replicates, data is mean ± SEM). No significant difference (ns) in PrP^res^ levels was observed, as assessed by an unpaired two-tailed *t* test (*p* = 0.79). *G*, cell immunoblots of CAD5(pIRESneo3) cells and CAD5(pIRESpuro3) cells after three passages following infection with RML prions and treatment with the indicated concentrations of G418 or puromycin. In *A* and *B*, undigested PrP blots were probed with the antibody HuM-D13, whereas blots of PrP^res^ were probed with HuM-P. Molecular weight markers indicate kDa.

G418 belongs to a larger family of aminoglycoside antibiotics that inhibit protein synthesis in predominantly Gram-negative bacteria. Unlike G418, other commonly used aminoglycosides like streptomycin and kanamycin are only effective against the bacterial ribosome. We wanted to see if these antibiotics, which are harmless to mammalian cells, could also inhibit *de novo* prion replication. Therefore, wild-type CAD5 cells were challenged with RML prions in the presence of concentrations of kanamycin or streptomycin that are equimolar to 1.0 mg/ml (1.4 mM) G418. Whereas PrP^res^ levels were decreased by ∼25% in cells cultured in the presence of kanamycin compared with untreated cells, prion infection was essentially blocked in cells cultured in the presence of streptomycin (Fig. 5, *A* and *B*). This indicates that other aminoglycoside antibiotics can hinder *de novo* prion infection in cultured cells to varying degrees. As the effect of streptomycin was so pronounced and given that streptomycin at 0.1 mg/ml is commonly added to cell culture medium to discourage bacterial growth, we assessed the effect of culturing cells in lower streptomycin concentrations on RML prion infection. While PrP^res^ levels in CAD5 cells treated with 1.0 mg/ml streptomycin were decreased by ∼70%, PrP^res^ levels were not significantly altered when 0.5 mg/ml streptomycin was used (Fig. 5, *C* and *D*).

Puromycin, an aminonucleoside antibiotic commonly used as a selectable marker, also inhibits protein synthesis. Unlike the aminoglycosides, puromycin disrupts protein synthesis by causing premature chain termination. We wanted to see if the effects of the aminoglycoside antibiotics were recapitulated by a structurally distinct but somewhat functionally similar antibiotic such as puromycin. CAD5 cells stably transfected with an empty vector encoding puromycin resistance (pIRESpuro3) were used in this experiment. Puromycin-resistant CAD5(pIRESpuro3) cells were challenged with RML prions in the presence of either 0 or 2 μg/ml puromycin, the latter being a common concentration used for selecting puromycin-resistant cell lines. Following three passages, cell lysates were analyzed for the presence of PrP^res^. We found that puromycin had no effect on the accumulation of PrP^res^ (Fig. 5, *E* and *F*). To further analyze how G418 and puromycin affect the accumulation of PrP^res^ immediately following prion infection, cell blots (45) were generated on RML-challenged CAD5(pIRESneo3) or CAD5(pIRESpuro3) cells that had been infected in the absence or presence of G418 or puromycin, respectively. G418 substantially reduced the proportion of PrP^res^-positive cells, whereas puromycin had no effect. (Fig. 5*G*). Thus, aminoglycoside, but not aminonucleoside, antibiotics interfere with *de novo* prion infection in cultured cells.

### G418 does not interfere with PrP^C^ expression or localization

**Figure 6:**
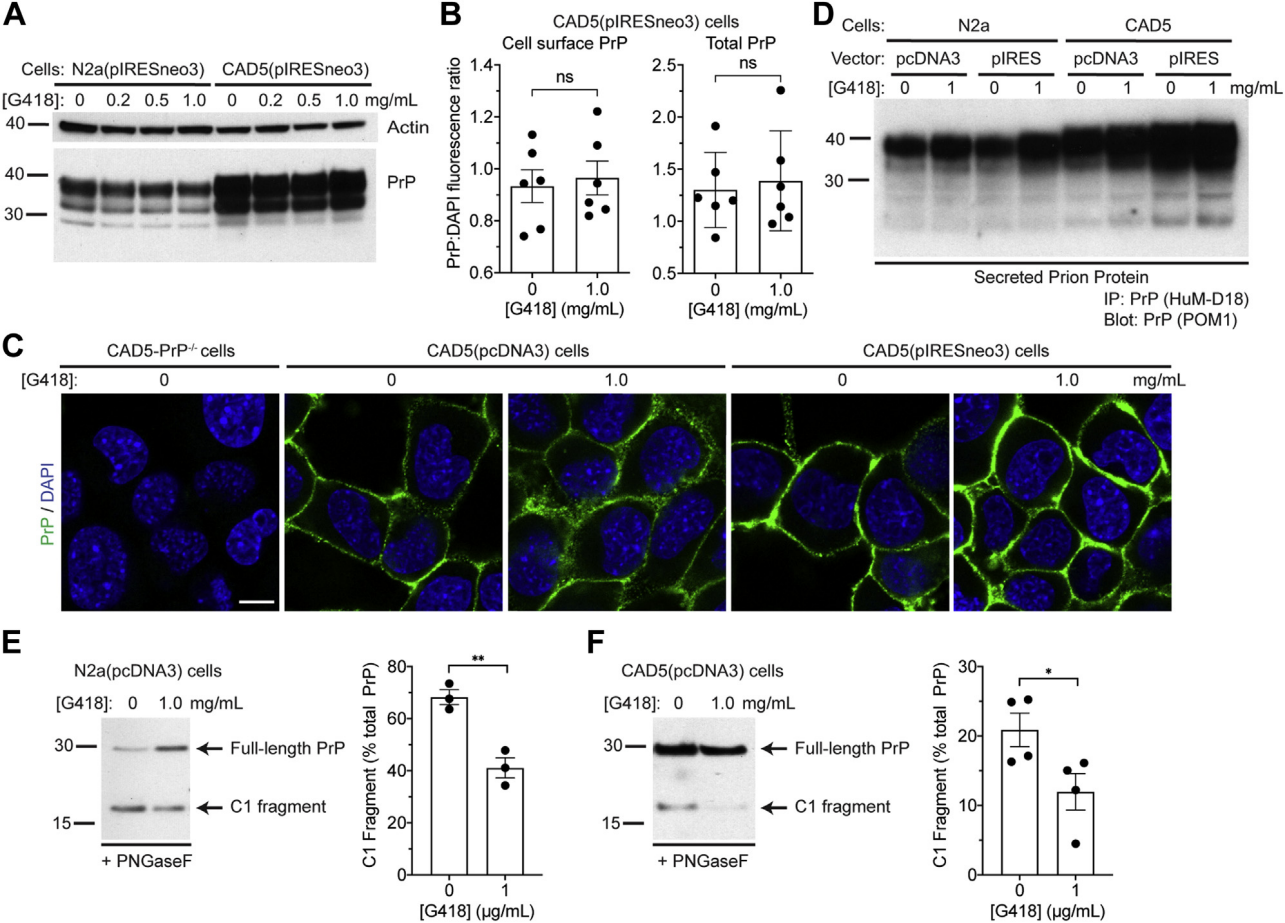
**G418 treatment does not interfere with PrP**^**C**^**expression or its cellular localization.***A*, immunoblot of PrP^C^ levels in N2a(pIRESneo3) and CAD5(pIRESneo3) cells that were treated with the indicated concentrations of G418 for 72 h. PrP was detected using the antibody HuM-D13. The blot was reprobed with an actin antibody to assess equal protein loading. *B*, quantification of full-length cell-surface and total PrP^C^ levels in CAD5(pIRESneo3) cells treated with the indicated G418 concentrations for 72 h using a microplate assay (data is mean ± SEM). Cells were stained with DAPI and the anti-PrP antibody HuM-D13, and then the PrP:DAPI fluorescence ratio was calculated. n = 6 independent biological replicates. No significant differences (ns) in cell-surface (*p* = 0.73) or total (*p* = 0.73) PrP^C^ levels were observed between untreated and G418-treated cells, as assessed by unpaired two-tailed *t* tests. *C*, immunofluorescence images of PrP^C^ in non-permeabilized CAD5-PrP^−/−^, CAD5(pcDNA3), and CAD5(pIRESneo3) cells that were left untreated or treated with 1 mg/ml G418 for 72 h. Cells were stained with DAPI (*blue*) and the anti-PrP antibody POM1 (*green*). Scale bar = 10 μM (applies to all images). *D*, immunoblot of secreted PrP levels in either N2a or CAD5 cells stably transfected with the pcDNA3 or pIRESneo3 vectors. Cells were either treated with 1 mg/ml G418 for 24 h or left untreated. Secreted PrP in the conditioned medium was isolated by immunoprecipitation with HuM-D18 and then detected using the antibody POM1. *E* and *F*, assessment of PrP^C^ cleavage in G418-treated cells. Immunoblots for PrP in PNGaseF-treated lysates as well as quantification of C1 fragment levels in N2a(pcDNA3) (*E*) and CAD5(pcDNA3) (*F*) cells that were treated with the indicated concentrations of G418 for 72 h (n = 3 or 4 independent biological replicates, respectively; data is mean ± SEM). ∗∗*p* = 0.0049 and ∗*p* = 0.047 by unpaired, two-tailed *t* tests. Full-length PrP and its C1 cleavage fragment were detected using the antibody HuM-D18. Molecular weight markers indicate kDa.

To probe the mechanism by which G418 might interfere with *de novo* prion infection, we tested its effect on PrP^C^ levels in G418-resistant CAD5 and N2a cells. Both lines were exposed to increasing concentrations of G418 for 72 h. No apparent change in PrP^C^ levels within cell lysates was detected in either line (Fig. 6*A*). To quantify cell surface and total PrP^C^ levels, cells were treated for 72 h in microplates, and then fluorescence was measured using a plate reader. When using an antibody that binds to residues 95 to 105 of PrP (HuM-D13), we found that both cell-surface and total PrP^C^ expression levels were similar following treatment with G418 (Fig. 6*B*). Next, we investigated whether the localization of PrP^C^ was altered by G418. CAD5(pcDNA3) and CAD5(pIRESneo3) cells were treated with G418 or left untreated and then fixed, immunolabeled for PrP, and visualized using confocal microscopy. The levels and distribution of PrP^C^ at the cell surface were not appreciably altered by G418 treatment (Fig. 6*C*). As expected, no PrP staining was observed in CAD5-PrP^−/−^ cells.

G418 and other aminoglycosides have been reported to partially influence the activity of phospholipases, resulting in increased shedding of GPI-anchored proteins into the media (46). Since PrP^C^ that has been shed into the media might bind to and sequester PrP^Sc^ seeds at the time of infection, thereby reducing the ability of PrP^Sc^ to interact with membrane-bound PrP^C^, we tested whether G418 modulates PrP^C^ shedding. Four different lines of G418-resistant CAD5 or N2a cells were treated with G418, and the conditioned media were collected 24 h later and then analyzed for PrP following immunoprecipitation. Levels of secreted PrP^C^ were only slightly increased in the G418-treated compared with the untreated samples (Fig. 6*D*), suggesting that G418 is unlikely to interfere with prion infection *via* an increase in PrP^C^ shedding. Finally, we tested whether proteolytic processing of PrP^C^ is affected by G418 treatment. A proportion of PrP^C^ normally undergoes endoproteolytic cleavage in the vicinity of residues 110/111 to produce a C1 fragment, which is believed to be unable to undergo conversion into PrP^Sc^ (47, 48, 49). In G418-resistant N2a and CAD5 cells, C1 cleavage of PrP^C^ was moderately but significantly reduced following treatment with G418 for 72 h (Fig. 6, *E* and *F*).

### Effects of antibiotics on the biophysical properties of recombinant PrP

**Figure 7:**
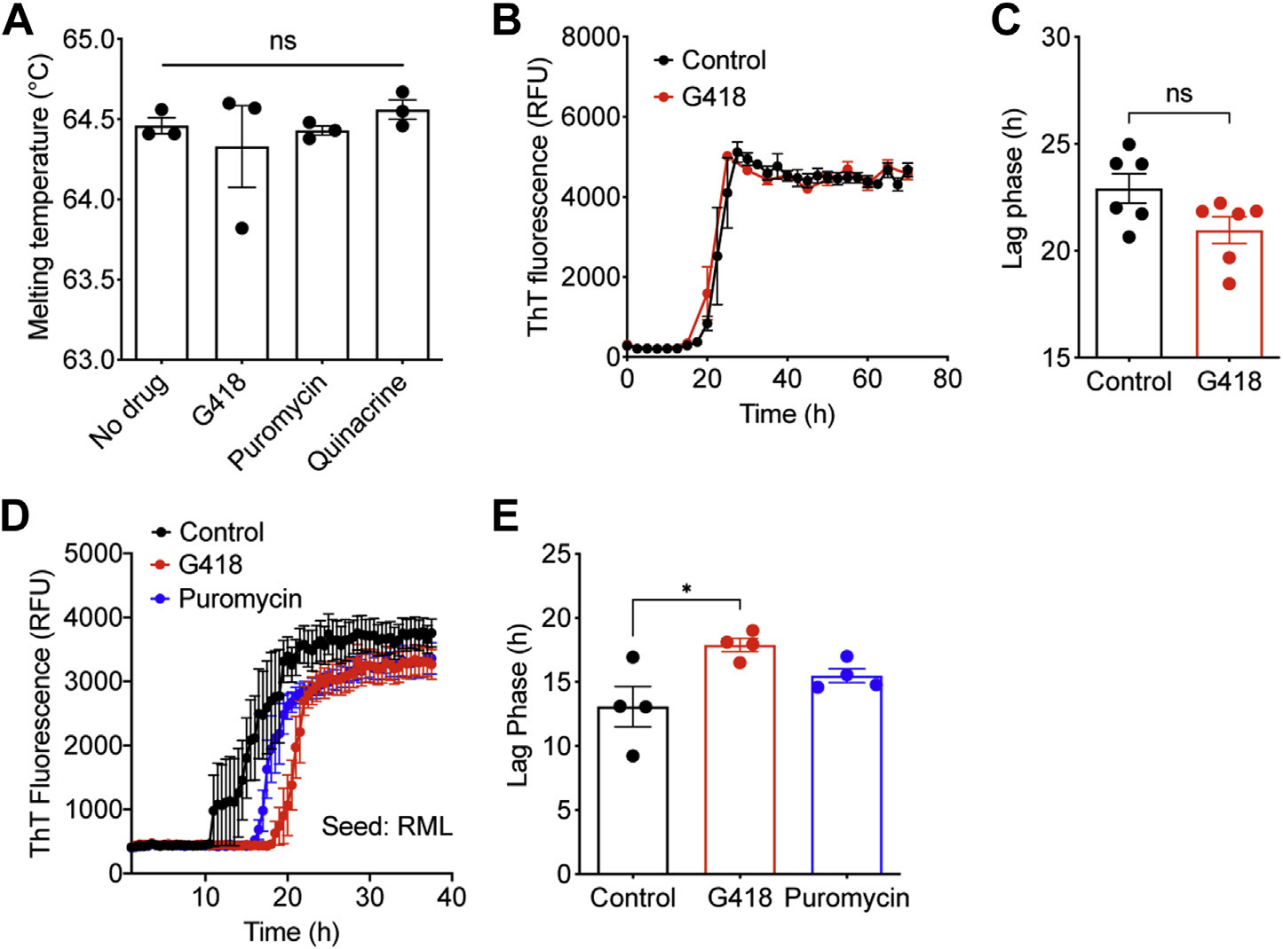
**G418 does not affect the stability or the aggregation of recombinant PrP.***A*, melting temperatures (mean ± SEM) of recombinant MoPrP (residues 23–230), as determined by differential scanning fluorimetry, in the presence or absence of a tenfold molar excess of either G418, puromycin, or quinacrine. n = 4 independent biological replicates; ns, not significant by one-way ANOVA (*p* = 0.69). *B*, ThT fluorescence curves for the spontaneous aggregation of recombinant MoPrP in the absence (*black*) or presence (*red*) of a tenfold molar excess of G418. Each data point represents the mean ± SEM of six independent biological replicates. *C*, calculated lag phases for the spontaneous aggregation of recombinant MoPrP in the absence (*black*) or presence (*red*) of a tenfold molar excess of G418. n = 6 independent biological replicates; ns, not significant by an unpaired two-tailed *t* test (*p* = 0.062). *D*, RT-QuIC analysis of RML-seeded recombinant MoPrP in the absence (*black*) or presence of a tenfold molar excess of either G418 (*red*) or puromycin (*blue*). Each data point represents the mean ThT fluorescence ± SEM of four independent biological replicates. *E*, lag phases (mean ± SEM) for RML-seeded RT-QuIC reactions using recombinant MoPrP in the absence (*black*) or presence of a tenfold molar excess of either G418 (*red*) or puromycin (*blue*). n = 4 independent biological replicates; ∗*p* = 0.015 compared with control reactions by one-way ANOVA followed by Dunnett’s multiple comparisons test.

To investigate whether G418 or puromycin might interact directly with PrP^C^, we generated recombinant MoPrP (recPrP) and performed differential scanning fluorimetry, a technique capable of detecting shifts in PrP thermal stability upon binding of small molecules (50, 51). In the presence of a tenfold molar excess of either puromycin, G418, or quinacrine, a ratio commonly used in protein thermal shift experiments (52), the melting temperature of recPrP remained unchanged, suggesting that none of these compounds likely act by binding to PrP^C^ and modulating its stability (Fig. 7*A*). Next, we tested whether G418 modulates the aggregation kinetics of recPrP using a Thioflavin T (ThT) fluorescence assay. A tenfold molar excess of G418 did not alter the lag phase for shaking-induced recPrP aggregation nor the shape of the aggregation curve, suggesting that G418 does not influence the spontaneous polymerization of recPrP (Fig. 7, *B* and *C*). An important caveat to this finding is that recPrP aggregates formed spontaneously *in vitro* are not highly infectious and are structurally distinct from PrP^res^ generated in a brain or cellular environment (53). Therefore, these results do not exclude the possibility that G418 may affect the spontaneous polymerization of membrane-anchored and glycosylated PrP^C^ *in vivo*. Finally, we tested the ability of puromycin and G418 to modulate the prion-induced templating of recPrP into aggregates using real-time quaking-induced conversion (RT-QuIC) (54). Both G418 and puromycin modestly increased the lag phase for RT-QuIC reactions when using RML prions as the seed (Fig. 7, *D* and *E*). Given that G418, but not puromycin, affects *de novo* prion infection, it seems unlikely that this small shift in lag phase is sufficient to explain the effects observed in cultured cells.

### Exposure to G418 does not lead to the selection of drug-resistant prions

**Figure 8:**
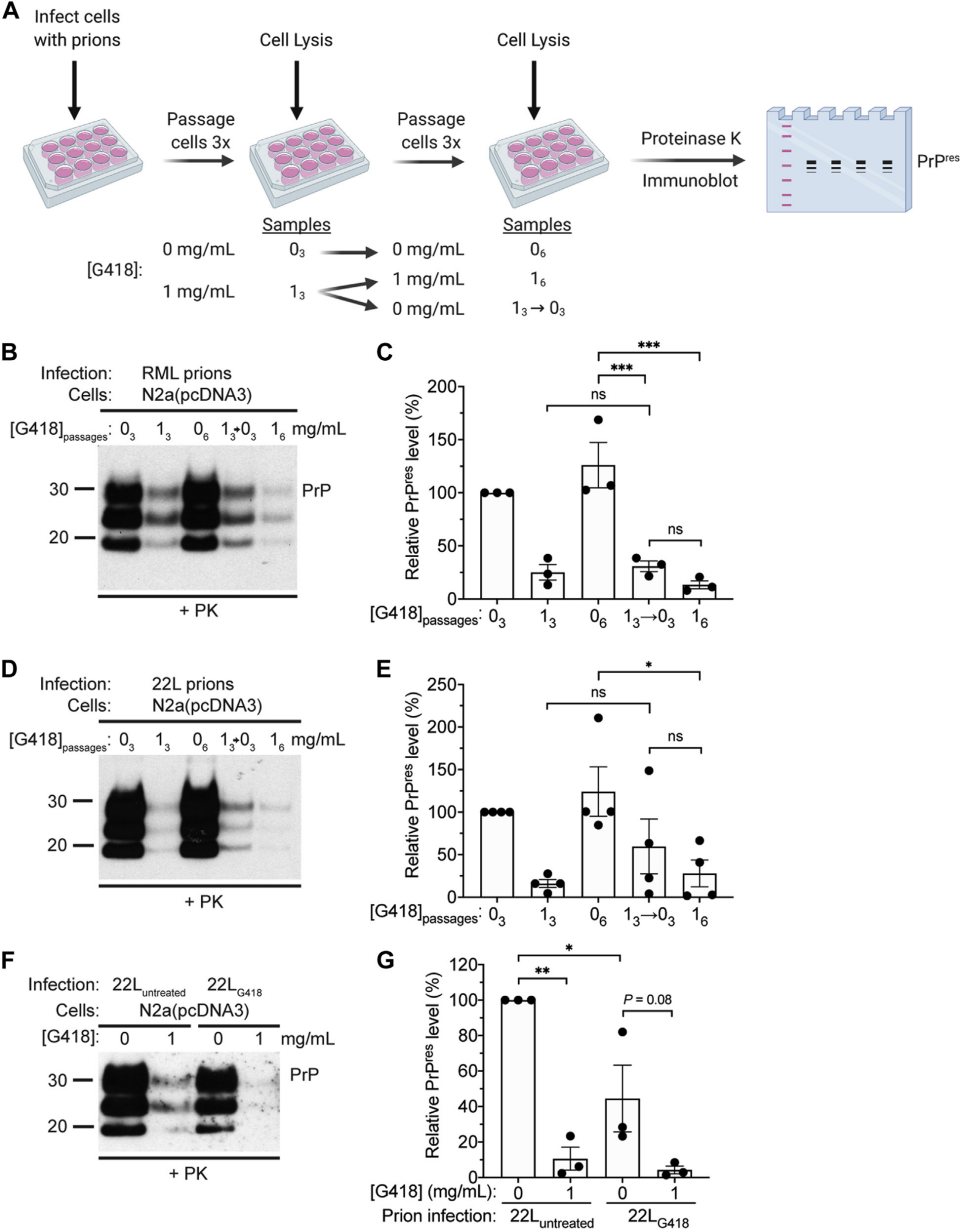
**G418 treatment does not lead to the selection of G418-resistant prions.***A*, schematic of the cellular infection experiment. *B*, immunoblot of PrP^res^ levels in RML prion-challenged N2a(pcDNA3) cells following three passages in the absence (0_3_) or presence (1_3_) of 1.0 mg/ml G418, six passages in the absence (0_6_) or presence (1_6_) of 1.0 mg/ml G418, or three passages in the presence of 1.0 mg/ml G418 followed by three passages in the absence of G418 (1_3_→0_3_). *C*, quantification of PrP^res^ levels in N2a(pcDNA3) cells challenged with RML prions in the presence or absence of 1.0 mg/ml G418 and cultured for the indicated the number of passage (n = 3 independent biological replicates, data is mean ± SEM). ∗∗∗*p* < 0.001 by one-way ANOVA followed by Tukey’s multiple comparisons test; ns, not significant. *D*, immunoblot of PrP^res^ levels in 22L prion-challenged N2a(pcDNA3) cells following three passages in the absence or presence of 1.0 mg/ml G418, six passages in the absence or presence of 1.0 mg/ml G418, or three passages in the presence of 1.0 mg/ml G418 followed by three passages in the absence of G418. *E*, quantification of PrP^res^ levels in N2a(pcDNA3) cells challenged with 22L prions in the presence or absence of 1.0 mg/ml G418 and cultured for the indicated the number of passage (n = 4 independent biological replicates, data is mean ± SEM). ∗*p* < 0.05 by one-way ANOVA followed by Tukey’s multiple comparisons test. *F*, immunoblot of PrP^res^ levels in N2a(pcDNA3) cells infected with prions from N2a(pcDNA3) cells (passage 6) that were infected with 22L prions either in the absence (22L_untreated_) or presence (22L_G418_) of 1.0 mg/ml G418. Cells were passaged three times in the absence or presence of 1.0 mg/ml G418. *G*, quantification of PrP^res^ levels in N2a(pcDNA3) cells infected with either 22L_untreated_ or 22L_G418_ prions in the presence or absence of 1.0 mg/ml G418 (n = 3 independent biological replicates, data is mean ± SEM). ∗∗*p* < 0.01, ∗*p* < 0.05 by one-way ANOVA followed by Tukey’s multiple comparisons test. Molecular weight markers indicate kDa.

To determine whether the decrease in PrP^res^ levels that occurs when cells are infected in the presence of G418 is reversible, we compared PrP^res^ levels in RML prion-challenged N2a(pcDNA3) cells following six passages in the presence or absence of G418 to cells in which G418 treatment was applied for three passages and then removed for the remaining three passages (Fig. 8*A*). As expected, PrP^res^ levels were strikingly reduced in cells exposed to G418 compared with untreated cells following either three or six passages (Fig. 8, *B* and *C*). However, there was no difference in PrP^res^ levels between cells exposed to G418 for three or six passages following infection and cells exposed to G418 for three passages followed by removal of the G418 for an additional three passages. Similar results were obtained when we repeated this experiment using the 22L strain as the source of prions (Fig. 8, *D* and *E*). Two possible explanations for the lack of PrP^res^ recovery following cessation of G418 treatment are: 1) the additional three passages following removal of G418 are insufficient to allow recovery of PrP^res^ to normal levels; 2) infection in the presence of G418 leads to the selection and propagation of a G418-resistant prion strain that accumulates to lower steady-state levels than the parental strain.

To evaluate whether G418 treatment at the time of prion infection leads to the propagation of a drug-resistant prion strain, we infected N2a(pcDNA3) cells with 22L prions in either the presence or absence of 1.0 mg/ml G418 and then passaged them six times. As expected, PrP^res^ levels were much lower in the cells exposed to G418 (Fig. 8, *D* and *E*). Cellular homogenates from 22L-infected untreated (22L_untreated_) or G418-treated (22L_G418_) cells were then used to infect naïve N2a(pcDNA3) cells, again in the presence or absence of G418. As predicted, G418 treatment reduced PrP^res^ levels in cells that were infected with 22L_untreated_ prions (Fig. 8, *F* and *G*). Due to the difference in PrP^res^ levels between 22L_untreated_ and 22L_G418_ at the time of infection (Fig. 8, *D* and *E*), prion accumulation in untreated cells was lower when 22L_G418_ prions were applied (Fig. 8, *F* and *G*). When 22L_G418_ prions were applied to cells, PrP^res^ levels were still substantially lower in cells infected in the presence of G418, although this did not reach statistical significance (Fig. 8, *F* and *G*). If G418 treatment had led to the selection and propagation of a G418-resistant prion strain, we would have expected to see similar PrP^res^ levels in G418-treated and untreated cells following infection with 22L_G418_ prions. Thus, G418 treatment is unlikely to result in the emergence of drug-resistant prions.

## Discussion

In this study, we investigated the inhibitory role of G418 and other aminoglycosides when attempting to infect antibiotic-resistant cultured cells with prions. We show that G418 inhibits *de novo* prion replication in a dose-dependent manner when present at the time of infection and during initial cellular passaging. This phenomenon was reproduced using distinct plasmid vectors, different murine cell lines, and two unique strains of mouse prions. Moreover, G418 also interfered with the infection of cells with hamster prions. This indicates that G418 hinders *de novo* prion infection in a fundamental way that applies across different PrP species and prion strains. We also found that another aminoglycoside antibiotic, streptomycin, can significantly inhibit the ability of cells to become infected with prions, although the inhibitory effect of streptomycin was only observed at concentrations exceeding those that would normally be used for discouraging microbial growth in cultured cells. Thus, the antibiotic composition of growth medium used when infecting cultured cells with prions needs to be carefully considered to maximize the efficiency of prion infection.

Infection of cultured PrP^−/−^ cells engineered to express PrP alleles of interest with prions constitutes a powerful paradigm for dissecting the molecular determinants of prion replication. Several strategies can be envisioned to eliminate or reduce the negative effects of G418 in such prion infection experiments. First, lower concentrations of G418 can be used to minimize the antiprion activity of G418 while still promoting the maintenance of PrP^C^ levels within a heterogeneous pool of cells. We found that PrP^C^ levels in polyclonal CAD5-PrP^−/−^ cell lines stably expressing MoPrP or HaPrP were directly correlated with the concentration of G418 present in the media. For both RML and 263K prion strains, infection of cells in the presence of 0.2 mg/ml G418 permitted prion replication despite the presence of lower PrP^C^ levels. Second, monoclonal lines of stably PrP-transfected PrP^−/−^ cells can be created to circumvent the genetic drift issue and remove the necessity for continuous presence of G418 in the culture medium. Finally, it may be possible to use a different selectable marker. In our experiments, puromycin had no effect on the ability of CAD5 cells with endogenous MoPrP expression to become infected with RML prions, although we do not know whether this is true for all prion strains. Additional caution should be exercised as puromycin treatment can lead to the emergence of puromycin-resistant subclones in aneuploid cell lines, even in cells that have not been transfected with a puromycin-resistance gene (55).

Other antibiotics have been documented to influence prion propagation in cells or animals. Treatment of prion-infected cells with the polyene antifungal agent amphotericin B reduces levels of PrP^res^ (56), and hamsters or mice treated with amphotericin B exhibit moderate survival extensions following prion infection (57, 58). Tetracycline and doxycycline have also been shown to decrease PrP^res^ levels *in vitro* and could extend the survival of prion-challenged animals (59, 60). However, neither amphotericin B nor doxycycline has proven to be effective at treating prion disease in humans (61, 62, 63). Despite its ability to hinder *de novo* prion infection in cultured cells, it is highly unlikely that G418 will be a viable treatment option for prion diseases. G418 inhibits protein synthesis in eukaryotic cells by blocking translational elongation and thus would be expected to have toxic effects following prolonged exposure in humans. It is possible, however, that other aminoglycosides or G418 derivatives might exist that could prove beneficial for treating prion disease.

The effects of G418 and other aminoglycosides on *de novo* prion infection in antibiotic-resistant cell lines are likely independent of their ability to disrupt protein synthesis since puromycin, which inhibits translation by an orthogonal method, had no discernible effect, whereas streptomycin, which has no effect on mammalian ribosomes, significantly hindered cellular prion infection. The differential activities of equimolar amounts of G418, streptomycin, and kanamycin in RML-challenged CAD5 cells (∼70%, ∼95%, and ∼25% reduction in PrP^res^ levels, respectively) further suggest that there is an element of specificity to the response. It is conceivable that the action of G418 and streptomycin may be related to their ability to permit read-through of premature termination codons and normal STOP codons (64, 65), possibly leading to enhanced production of a protein that interferes with *de novo* prion infection. Indeed, G418-induced translational errors have been shown to modulate the production of the yeast prion [URE3] (66). However, the precise mechanism by which G418 hinders *de novo* prion infection in cultured cells remains enigmatic. At the present time, we know more about the cellular mechanisms relevant to prion infection that remain unaffected by G418 treatment. G418 treatment had no discernible effects on PrP^C^ levels, PrP^C^ cell surface localization, the thermal stability of recPrP, or the spontaneous formation of recPrP aggregates. Collectively, these results suggest that G418 is unlikely to act by binding to PrP^C^ and/or by influencing its biogenesis. A moderate decrease in the extent of PrP C1 cleavage was observed following G418 treatment. However, if anything, this should result in an increased amount of PrP^res^ since the PrP C1 fragment has been proposed to be a dominant-negative inhibitor of prion replication (47). We also saw no evidence that G418 treatment alters strain properties in PrP^Sc^, including the glycoform ratio in PrP^res^ and its molecular weight. Moreover, G418 did not lead to the emergence of a novel drug-resistant prion strain, as has been observed following treatment with other antiprion small molecules (22, 67, 68, 69). Finally, it is unlikely that G418 treatment resulted in the emergence of protease-sensitive prions (70, 71). It has been shown that PK-resistant and PK-sensitive preparations possess similar levels of infectivity and lead to identical amount of PrP^res^ being produced upon injection into hamsters (71). Infecting cells with homogenates from cells that were previously infected with 22L prions in the presence of G418 resulted in lower levels of PrP^res^ than when using homogenates from cells that were not exposed to G418 (Fig. 8*G*). This suggests that G418 treatment during *de novo* cellular prion infection leads to a reduction in PrP^res^ levels and a corresponding decrease in prion infectivity, implying that G418 treatment does not increase levels of PK-sensitive prions.

Despite having a pronounced inhibitory effect on *de novo* prion infection in cultured cells, G418 had little to no effect on PrP^res^ levels in cells with established prion infection. While there is no shortage of small molecules capable of reducing or eliminating PrP^res^ in cultured cells infected with mouse prions (20, 72), to the best of our knowledge, G418 is the only drug that selectively exerts its influence during the initial phases of cellular infection. We envision two scenarios that may explain the selective effect of G418 on *de novo* cellular prion infection compared with established cellular prion infection:

### G418 may specifically interfere with prion replication by blocking a PrP^Sc^ species that is selectively present in the prion inocula

To generate the source of prions used for the cellular prion infection experiments, we homogenized cells using a bead beater apparatus. During this process, smaller PrP^Sc^ species may be generated, which are known to be more infectious (73), and it is conceivable that G418 may selectively act on smaller PrP^Sc^ assemblies. Consistent with this theory, the presence of G418 significantly delayed the seeded formation of recPrP aggregates in an RT-QuIC assay, potentially suggesting that G418 might partially interfere with an interaction between PrP^C^ and PrP^Sc^. However, these results should be interpreted with caution since puromycin, which had no effect in a cellular prion infection assay, also slightly delayed the seeded formation of recPrP aggregates. A candidate PrP^Sc^ species that may be selectively present in prion inoculum preparations is nonanchored PrP^Sc^. In persistently infected cells, it has been determined that cell–cell contact, possibly *via* tunneling nanotubes, or mother-to-daughter transmission during cell division are the primary mechanisms responsible for the spreading and accumulation of prion infection (74, 75, 76). While PrP^Sc^ can also be released into the medium of cultured cells, perhaps *via* exosomes, infection *via* cell medium is not as efficient (74, 77). Prion replication *via* cell–cell contact or vertical transmission would both involve an interaction between membrane-anchored PrP^C^ and PrP^Sc^, whereas transmission through the cell medium, which is more akin to the procedure used to infect cells *de novo*, is more likely to involve PrP^Sc^ species that have been released from the membrane. G418 may be better at blocking prion replication when PrP^Sc^ is not constrained to the plasma membrane.

### G418 may sensitize cells to a toxic PrP species that is only present in the initial stages of *de novo* cellular prion infection

Cytopathic effects have not been commonly documented in prion-infected immortalized cell lines, suggesting that cells with established prion infection do not produce highly toxic PrP species. However, it has been hypothesized that neurotoxic PrP species catalyzed by an interaction between PrP^Sc^ and PrP^C^ are responsible for neuronal dysfunction and death in mice and cultured primary neurons (78, 79, 80). Such toxic species may be transiently generated during the initial stages of cellular prion infection, possibly due to a specific type of PrP^Sc^-PrP^C^ interaction that does not occur in chronically infected cells. The toxicity of this PrP species may be enhanced by G418, and this could lead to a selective elimination of cells with high levels of PrP^res^. Interestingly, cells expressing a neurotoxic PrP mutant lacking the hydrophobic tract (PrPΔ105–125) are more rapidly eliminated following G418 treatment than cells expressing wild-type PrP (81, 82), which may be related to the formation of pores in the membrane and/or induction of spontaneous ion currents (83, 84). Thus, G418 may be having a similar effect on cells during *de novo* prion infection.

Our findings reveal that the negative effects of G418 and other antibiotics need to be considered when trying to establish accurate and efficient cellular models of prion replication. The cellular determinants of prion susceptibility in cell culture remain largely unknown, and therefore it is prudent to be diligent about the factors present during the infection and propagation phases of cellular prion infection. On the other hand, molecules such as G418 may also provide a unique opportunity to dissect early events during prion infection. New insight into this process may yield novel strategies for blocking the earliest stages of prion replication during prion disease.

## Experimental procedures

### Chemicals

G418 sulfate powder (Geneticin) was purchased from either Thermo Fisher Scientific (#11811031), BioShop (#GEN418.5), or FroggaBio (#400-111P-5G). Puromycin dihydrochloride (#PUR333.100), kanamycin sulfate (#KAN201.10), and streptomycin sulfate (#STP101.25) were obtained from BioShop. All cell culture reagents were purchased from Thermo Fisher Scientific unless otherwise indicated.

### Cell culture

Mouse Neuro2a (N2a) neuroblastoma cells (ATCC# CCL-131) were cultured in DMEM medium (Thermo Fisher #11965118) containing 10% (v/v) FBS (Thermo Fisher #12483020), 1× GlutaMAX (Thermo Fisher #35050061), and 0.2× penicillin-streptomycin (Thermo Fisher #15140122). Mouse CAD5 catecholaminergic and CAD5-PrP^−/−^ (clone D6) cells (34) were cultured in Opti-MEM medium (Thermo Fisher #31985088) containing 5 to 10% (v/v) FBS, 1× GlutaMAX, and 0.2× penicillin-streptomycin. All cell lines were kept at 37 °C in a 5% CO_2_ incubator. CAD5-PrP^−/−^ cells were generated previously using CRISPR/Cas9 gene editing technology (34, 85). N2a cells were passaged at 1:10 dilution every 2 to 3 days using 0.25% trypsin-EDTA solution (Thermo Fisher #25200056). CAD5 cells were passaged at 1:5 dilution every 4 to 5 days using an enzyme-free cell dissociation reagent (Millipore #S-014-B).

### Generation of stable cell lines

The open reading frames of either mouse or Syrian hamster PrP were inserted into the vectors pcDNA3 (Thermo Fisher), pIRESneo3 (Clontech), or pIRESpuro3 (Clontech). pcDNA3 and pIRESneo3 both confer resistance to G418, whereas pIRESpuro3 confers resistance to puromycin. In pIRESneo3 and pIRESpuro3, expression of the antibiotic resistance gene is coupled to expression of a gene of interest through use of an internal ribosome entry site (IRES). In pcDNA3, expression of the gene of interest and the antibiotic resistance gene are controlled by distinct promoters. For cloning into pcDNA3, the *BamHI* and *XbaI* restriction sites were used, whereas *NheI* and *BamHI* sites were used for the pIRESpuro3 and pIRESneo3 plasmids. For generation of stably transfected cells, CAD5, CAD5-PrP^−/−^, or N2a cells were plated in a 6-well plate at a density of 5 to 7 × 10^5^ cells/well. These cells were then transfected with either empty vector or a PrP-encoding plasmid using Lipofectamine-2000 at a 2 μg plasmid DNA:4 μl Lipofectamine ratio in Opti-MEM medium. The next day, the cells were passaged into the appropriate medium containing either 1 mg/ml G418 or 2 μg/ml puromycin in 10 cm tissue culture dishes. The selection was conducted over 2 to 3 weeks, after which G418-resistant cells were maintained in 0.2 mg/ml G418. All stable cell lines constitute polyclonal pools of cells; limiting dilution subcloning was not performed.

### Prion strains

Mouse RML and 22L prion inocula were obtained from RML- or 22L-infected CAD5 cells (34). The cells were grown to confluency, scraped in 1 ml PBS per 10 cm dish, and then homogenized using a Minilys bead homogenizer and CK14 homogenization tubes (Bertin). Benzonase (50 units/ml; EMD Millipore #70746-4) was added into the scraped culture, and then the culture was homogenized for three cycles of 30 s at maximum speed, with 5 min of incubation on ice between each cycle. Cell homogenates were then stored at −80 °C. Syrian hamster 263K prion inoculum was prepared in a similar way from 263K-infected CAD5-PrP^−/−^ cells stably expressing HaPrP (34).

### Cellular prion infections

For all experiments involving prion-infected cells, penicillin-streptomycin was omitted from the medium. Cells to be infected with prions were plated 24 h prior to infection in a 12-well dish at 1.5 × 10^5^ cells/well or 3 × 10^5^ cells/well for N2a and CAD5 cells, respectively. Prion-containing cellular homogenates were quantified using a bicinchoninic acid (BCA) assay, and then 100 μg of each homogenate was diluted in the appropriate growth medium and added onto cells, which were 60 to 70% confluent at the time of infection. Aminoglycoside or puromycin antibiotics were added to the diluted cellular homogenate at the desired concentration at the time of infection to prepare the final inoculum. Cells were exposed to the inoculum for 72 h and then passaged in the presence or absence of antibiotics for three passages. At this point, the cells were split off in 6-well plates or 6-cm dishes and lysed as described below. To generate the ScCAD5 cells, CAD5 cells stably transfected with empty vector were infected with RML prions and then passaged a minimum of 3 to 6 times in the absence of G418.

### Cell lysis and immunoblotting

Cells were washed twice in PBS and then lysed in lysis buffer [50 mM Tris-HCl pH 8, 150 mM NaCl, 0.5% (v/v) NP40 and 0.5% (w/v) sodium deoxycholate] containing Halt protease inhibitor cocktail (Thermo Fisher #87786). The lysates were then incubated on ice for 15 min, with 30 s of vortexing every 5 min. The samples were then centrifuged at 1000*g* for 5 min to remove debris. The lysates were quantified using a BCA assay, and then the desired amount of protein was prepared in 1× Bolt LDS loading buffer (Thermo Fisher #B0007) containing 2.5% (v/v) β-mercaptoethanol. Samples were then boiled and loaded onto 10% or 4–12% Bolt Bis-Tris gels (Thermo Fisher) for SDS-PAGE. Following gel electrophoresis, proteins were transferred onto Immobilon-P membranes using Tris-Glycine transfer buffer (137 mM Glycine, 100 mM Tris-HCl pH 8). Membranes were then incubated in blocking buffer [Tris buffered saline (TBS) with 5% skim milk and 0.05% (v/v) Tween-20] for 1 h. The blocked membranes were then incubated in primary antibodies overnight at 4 °C. The next day, the membranes were washed three times with TBST [TBS +0.05% (v/v) Tween-20] for 10 min each. Secondary antibodies conjugated with horseradish peroxidase (BioRad) were then added to the membranes in blocking buffer on a rocker for 1 h at room temperature. The blots were again washed three times with TBST, for 10 min each. Following these washes, the blots were developed using Western Lightning ECL Pro (PerkinElmer), and exposed to HyBlot CL x-ray film. PrP antibodies used in this study were either recombinant humanized Fabs, including HuM-D18 (1:5000 dilution), HuM-D13 (1:5000 dilution), and HuM-P (1:10,000 dilution) (86, 87), or the mouse monoclonal antibody POM1 (1:2000 dilution; MilliporeSigma #MABN2285) (88). Actin blots were conducted on the previously probed blots following inactivation of horseradish peroxidase using 0.05% (w/v) sodium azide. Following washes with TBST, the blots were probed with the Actin 20–33 antibody (1:10,000 dilution; Sigma Aldrich #A5060) in blocking buffer. Immunoblots were scanned and then densitometry was performed using ImageJ.

### Enzymatic digestions

For experiments involving prion-infected cells, protease inhibitor was omitted from the cell lysis buffer. Cell lysates were quantified using a BCA assay and volumes adjusted with lysis buffer to achieve identical protein concentrations. Then, 0.5 to 1 mg of lysate was digested with proteinase K (PK) (Thermo Fisher Scientific #EO0491) at 50 μg/ml for 1 h at 37 °C with shaking at 600 rpm. The PK:protein ratio was kept at 1:50 to allow for rigorous digestion of PrP^C^. The reactions were stopped by the addition of PMSF to a final concentration of 2 mM, and the samples were then mixed with sarkosyl (Sigma Aldrich #61747) to a final concentration of 2% (v/v). The digested samples were ultracentrifuged at 100,000*g* for 1 h at 4 °C using a TLA-55 rotor (Beckman Coulter). The supernatant was discarded, and the pellet was resuspended in 30 μl of 1× Bolt LDS loading buffer containing 2.5% (v/v) β-mercaptoethanol. The samples were boiled for 10 min and then analyzed by immunoblotting. To analyze PrP C1 cleavage, cell lysates were deglycosylated for 4 h at 37 °C using PNGase F (New England BioLabs #P0704S) according to the manufacturer’s protocol. To quantify the PrP-C1 fragment, levels of C1 were expressed as a percentage of total PrP signal (C1 fragment + full-length PrP).

### Cell blotting

Cell blotting experiments were done as previously described (45). Cells were plated at 6 × 10^5^ cells/well in a 6-well dish, which contained 2 to 3 circular coverslips of 1 cm diameter, and then infected and passaged in the presence or absence or antibiotics as described above. The cells were grown to confluency and then an appropriately sized nitrocellulose membrane was activated in sterile dH_2_O and soaked in lysis buffer. The coverslips were removed and placed onto a filter paper soaked in lysis buffer. The soaked membrane was pressed against the coverslips firmly for 30 s. The coverslips were carefully removed from the membrane and discarded. The membrane was air dried and then stored at −20 °C until further use. The membranes were activated in lysis buffer and then subjected to PK digestion (5 μg/ml) in lysis buffer for 2 h at room temperature. The PK was inactivated with 2 mM PMSF and then the membrane was washed three times with dH_2_O followed by treatment with 4 M guanidine hydrochloride for 10 min to denature PrP^Sc^. The membrane was washed three times in dH_2_O and was then further processed using the immunoblotting protocol described above.

### Immunofluorescence

For quantitative immunofluorescence, CAD5 cells stably transfected with empty pIRESneo3 vector were plated at 12,000 cells/well in a 96-well plate. One day after plating, the cells were exposed to either 0 or 1 mg/ml G418 in quadruplicates. After 48 h, the cells were fixed with 4% (v/v) paraformaldehyde for 15 min at room temperature. The cells were then washed 1× with phosphate-buffered saline (PBS) and then either permeabilized with 0.1% (v/v) Triton X-100 in PBS for 10 min to detect total PrP or left untreated for the detection of cell-surface PrP. The cells were washed again with 1× PBS and then blocked for 1 h with 2% (v/v) goat serum (in 1× PBS). The cells were then incubated with the anti-PrP antibody HuM-D13 at a dilution of 1:500 overnight at 4 °C. The next day, the cells were washed 2× and then incubated with a goat anti-human AlexaFluor 488-conjugated secondary antibody (Thermo Fisher) at 1:500 in blocking solution (2% goat serum/PBS) for 1.5 h at room temperature. The cells were then washed 2× in PBS and then incubated with 4′,6-diamidino-2-phenylindole (DAPI) at 1:1000 dilution in PBS for 10 min. At this point, the 96-well plate was analyzed for AlexaFluor 488 (PrP^C^) fluorescence (excitation: 488 ± 7 nm; emission: 535 ± 15 nm) and DAPI fluorescence (excitation: 405 ± 10 nm; emission: 460 ± 15 nm) using the BMG CLARIOstar plate reader set at 1500 gain. Cellular PrP^C^ expression levels were calculated using the PrP^C^/DAPI signal ratio to provide a quantitative readout. For qualitative immunofluorescence, 1 × 10^5^ cells were plated in 24-well plates with #1.5 glass-like coverslip bottoms (Cellvis #P24-1.5P), which were then subjected to treatment with 0 or 1 mg/ml G418 for 48 h. Cells were fixed with 4% (v/v) paraformaldehyde for 15 min, blocked, and then PrP^C^ was detected using the antibody POM1 at a dilution of 1:500. Cells were washed and stained with DAPI as described above and then imaged using a Zeiss LSM880 confocal microscope.

### Analysis of secreted PrP^C^

CAD5 or N2a cells stably transfected with empty pcDNA3 or pIRESneo3 vectors were plated in a 6-well plate at 5 × 10^5^ cells/well in growth media lacking G418. One day later, the cells were treated with Opti-MEM media containing 0 or 1 mg/ml G418 for 24 h, and then the conditioned media was collected. The conditioned medium was centrifuged at 1500*g* to remove cellular material. Secreted PrP was immunoprecipitated from the conditioned medium using the antibody HuM-D18 as follows. KappaSelect resin (Cytiva #17545801) was used as the resin for the conjugation of HuM-D18. The resin was washed 3× in PBS and then conjugated with 5 μg of HuM-D18 antibody overnight at 4 °C with end-over-end rotation. The following day, the conjugated resin was washed 3× and then added to the conditioned medium and then left overnight at 4 °C with end-over-end rotation. The next day, the resin was washed 3×, and then eluates were obtained by boiling the resin in 1× Bolt LDS sample buffer for 10 min. The eluates were then analyzed by immunoblotting using the POM1 antibody for detection of PrP.

### Real-time quaking-induced conversion (RT-QuIC)

Recombinant MoPrP(23–230) was generated as described previously for recombinant Syrian hamster PrP (34). ScCAD5 cells were homogenized as described above, and then protein levels in homogenates quantified using the BCA assay. RT-QuIC was carried out as described previously, with conditions optimized for detection using MoPrP as the substrate (89). Briefly, RT-QuIC substrate mixture was prepared as follows: 0.1 mg/ml (4 μM) recombinant MoPrP(23–230), PBS (130 mM NaCl, 10 mM sodium phosphate pH 7.3), 10 μM ThT, and 1 mM EDTA. Prior to addition to the reaction mixture, recombinant MoPrP was centrifuged at 21,000*g* for 15 min to remove any preexisting aggregates. A tenfold molar excess (40 μM) of either G418 or puromycin was added where indicated. Ninety eight microliters of this reaction mixture was added to each well of a black, clear bottom 96-well plate (Nunc). Then, 2 μl of the seed mixture [PBS containing 0.05% (w/v) SDS, 1× N-2 Supplement (ThermoFisher), and 10 ng of ScCAD5 homogenate] was added to each well. The plate was sealed and then incubated at 42 °C in a BMG CLARIOstar microplate reader. The plate was subjected to alternating cycles of 1 min shaking (700 rpm, double orbital) and 1 min rest. ThT fluorescence (excitation: 444 ± 5 nm; emission: 485 ± 5 nm) was measured every cycle (every 2 min) for up to 38 h total using a gain setting of 1600.

### ThT aggregation assay

Recombinant MoPrP(23–230) was dialyzed to pH 7.4 in 10 mM sodium phosphate buffer. Following dialysis, reaction mixture was prepared using 10 mM sodium phosphate pH 7.4, 0.1 mg/ml (4 μM) MoPrP(23–230), 10 μM ThT, and 135 mM NaCl. A tenfold molar excess of G418 (40 μM) was added to the mixture where indicated. One hundred microliters of this mixture was pipetted to the bottom of each well of a black, clear bottom 96 well plate (Nunc). The plate was sealed and then subjected to continuous shaking (700 rpm, double orbital) for up to 80 h at 37 °C in a BMG CLARIOstar microplate reader. ThT readings (excitation: 444 ± 5 nm; emission: 485 ± 5 nm) were taken every 5 min. Lag phases for aggregation were calculated by fitting the kinetic curves to a sigmoidal dose–response (variable slope) model in GraphPad Prism, and then using the equation T_50_ − [1/(2∗k)], where k is the Hill slope and T_50_ is the time at which fluorescence is halfway between the baseline and plateau values (90).

### Differential scanning fluorimetry

Recombinant MoPrP(23–230) at a concentration of 0.1 mg/ml (4 μM) was incubated with SYPRO Orange according to the manufacturer’s instructions (ThermoFisher #4461146). The temperature was increased in slow 2 °C/min increments using a quantitative PCR machine (Light Cycler 480, Roche), and the fluorescence from the SYPRO orange dye was measured over time. Increased binding of the dye is directly proportional to exposure of hydrophobic residues as the protein becomes denatured. Where indicated, the reaction mixture contained a tenfold molar excess (40 μM) of either quinacrine, puromycin, or G418. Melting temperatures were calculated using Protein Thermal Shift software (Roche).

### Statistical analysis

In prion infection experiments comparing the means of two experimental conditions where all replicate samples were run together on the same gel, an unpaired two-tailed *t* test was used. In prion infection experiments where experimental replicates were run on different gels, samples were normalized to their respective control replicates, which were set at 100%, and then a one sample *t* test was performed. When greater than two experimental conditions were compared, a one-way ANOVA was used followed by Dunnett’s (when samples were compared with a defined control) or Tukey’s (when all samples were compared with each other) multiple comparisons test. The distribution of the data was assumed to be normal, but this was not formally tested. All statistical analysis was performed using GraphPad Prism software (version 9.0.1) with a significance threshold of *p* < 0.05.

## Data availability

All data are contained within the manuscript.

## Supporting information

This article contains supporting information.

## Conflict of interest

The authors declare that they have no conflicts of interest with the contents of this article.
