# Supplementary material for: The aminoglycoside G418 hinders de novo prion infection in cultured cells
Source: J Biol Chem. 2021 Aug 12;297(3):101073. doi: 10.1016/j.jbc.2021.101073 (PMC8413896; doi:10.1016/j.jbc.2021.101073)
Supplement: Supplemental Figure S1 [file mmc1.pdf]

Supporting Information: The aminoglycoside G418 hinders *de novo* prion infection in cultured cells

Hamza Arshad, Zeel Patel, Mohadeseh Mehrabian, Matthew E.C. Bourkas, Zaid A.M. Al-Azzawi, Gerold Schmitt-Ulms, and Joel C. Watts

This document contains: Figure S1

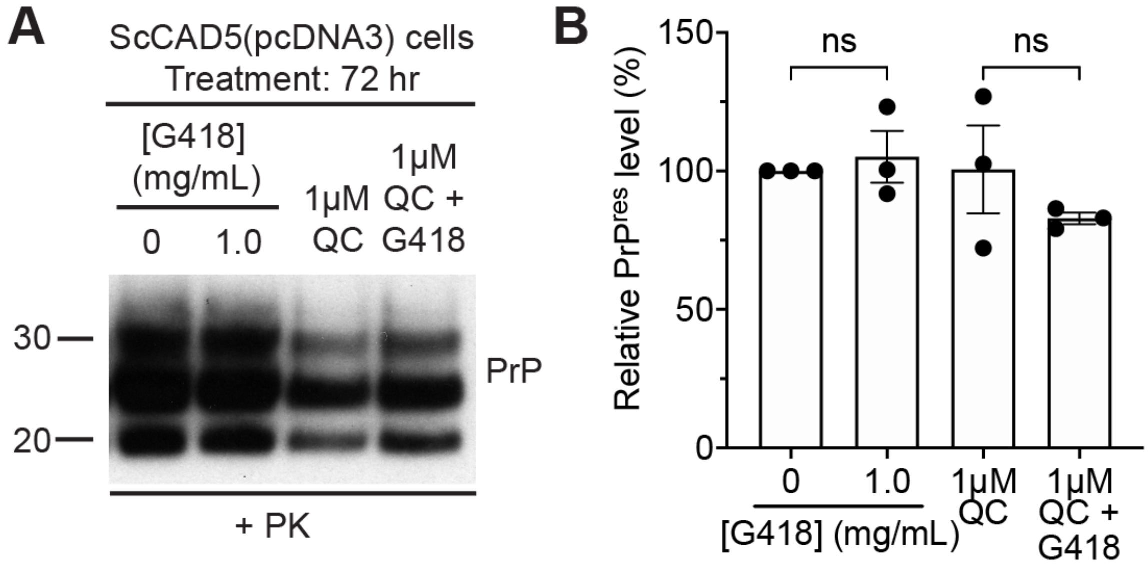

Figure S1. Treatment of RML prion-infected cells with G418 and 1 μM quinacrine. A Immunoblot of PrP^{res} levels in ScCAD5(pcDNA3)] cells treated with the indicated concentrations of G418, 1 μM QC, or 1 μM QC + 1.0 mg/mL G418 for 72 hours. PrP^{res} was detected using the antibody HuM-P. Molecular weight markers indicate kDa. **B** Quantification of PrP^{res} levels in ScCAD5(pcDNA3) cells following treatment with G418, QC, or QC + G418 for 72 hours. n = 3 independent biological replicates (data is mean ± SEM). PrP^{res} levels were not significantly different (P = 0.56) in cells treated with 1 μM QC or 1 μM QC + 1.0 mg/mL G418 as determined by one-way ANOVA followed by Tukey's multiple comparisons test.
